# Mobile phone addiction and its association with burnout in Chinese novice nurses: A cross‐sectional survey

**DOI:** 10.1002/nop2.673

**Published:** 2020-11-03

**Authors:** Huan Ma, Ji‐Qun He, Jin‐Mei Zou, Ying Zhong

**Affiliations:** ^1^ Xiangya Nursing School Central South University Changsha China; ^2^ Xiangya Hospital of Central South University Changsha China; ^3^ Nursing Department Sichuan Vocational College of Health and Rehabilitation Zigong China

**Keywords:** burnout, mobile phone addiction, novice nurse, nursing

## Abstract

**Aim:**

To explore the levels of mobile phone addiction and burnout and their relationships among novice nurses.

**Design:**

A cross‐sectional investigation design.

**Methods:**

Questionnaires were distributed to 400 novice nurses in five public hospitals in China. A total of 366 participants completed the survey. Data collected in 2020 were analysed by using descriptive statistics, an independent *t* test and Pearson's correlation analysis.

**Results:**

The results showed that the frequency of nursing adverse events was associated with higher level of mobile phone addiction in novice nurses, and 52.46% of the participants (*N* = 366) presented a high level of occupational burnout. Moreover, the results indicated that there was a positive correlation between the novice nurses’ mobile phone addiction level and burnout (*r* = .33, *p *< .01).

**Conclusion:**

The level of mobile phone addiction may affect nursing adverse events and nurses’ burnout. Education on novice nurses’ mobile phone use seems necessary to ensure patient safety and burnout prevention.

**Impact:**

Findings of this study expanded important knowledge about mobile phone addiction and its potential influence on nursing safety and nurse burnout and may place significant implications to staff nurse management and in‐service education.

## 1 INTRODUCTION

In China, the Internet users on mobile phones have climbed to 847 million, accounting for 99.1% of the overall network users by 2019. Mobile phone, as the main social network platform and driving force, is becoming more and more important for people in daily life by providing the users plenty of information, communication, education and entertainment through various mobile applications. Accessibility, usefulness, multi‐tasking and portability are their essential characters (Cho et al., 2016; Haug et al., 2015). Meantime, mobile phones offer the access to the Internet for the healthcare personnel with substantial potential advantages (Dennison et al., 2013). However, mobile phone overuse also has a negative effect on the patient safety and mental health of medical workers (Dante et al., 2016; Thomée, 2018). Furthermore, based on the data of the annual Top 10 Technology Hazards published by the Emergency Care Research Institute in 2013, “Caregiver distractions from mobile phones” was the ninth place in the ranking of health technology hazards(ECRI, 2012). Novice nurses are in the transition from student to competent registered nurses and undertake the important task of caring for patients. The special group has become a vital force of the nursing contingent in public hospitals. But the extent of this issue in novice nurses is still unknown. Consequently, it is necessary to conduct the empirical exploration on whether the level of mobile phone addiction associates with the incidence of nursing adverse events and burnout among novice nurses.

## 2 BACKGROUND

Mobile phone addiction has been described as a “problematic mobile phone use,” called “smartphone addiction,” “mobile phone dependence,” “cell phone overuse” or “no‐mobile‐phone‐phobia” (Mak et al., 2014). Smartphone overuse will do harm to individuals’ professional and family life and has been considered as potentially addictive. Currently, the study of mobile phone addiction has become a burning issue in the field of public health in many countries but most studies were conducted among junior and high school students, university students and medical students as their subjects, but few focused on the novice nurses (Demirci et al., 2015; Ibrahim et al., 2018; Kawyannejad et al., 2019; Lee et al., 2017; Lopez‐Fernandez et al., 2014; Parashkouh et al., 2018; Sahın et al., 2013). Novice nurses are fully involved in clinical nursing work and crucial for the recovery and safety of patients. Previous studies have shown that attention distraction of healthcare providers due to overuse and abuse of mobile phone in clinical care setting may cause nursing adverse events that will threaten the safety of patients (Alsos et al., 2012; Cohen et al., 2018; Katz‐Sidlow et al., 2012). However, whether there is a difference between the frequency of nursing adverse events and mobile phone addiction among novice nurses is still unknown. In addition, for individual nurses, the excessive use of mobile phone may affect their physical and mental health such as behaviour disorders, sleep disorder, depression and neck pain symptom in daily life (Elhai et al., 2017; Lee et al., 2015).

Burnout is a psychological syndrome and identified by three different factors including emotional exhaustion, depersonalization and personal accomplishment (Maslach et al., 2016). It occurs when employees work on high‐stress jobs with heavy workload and long working hours (Boamah et al., 2016). Burnout has been widely studied, especially the potential negative consequences it may bring to patients. For instance, a systematic review (Rodrigues et al., 2017) found that high level burnout syndrome may lead to unsafe care outcomes. Galletta et al. (2016) also revealed that burnout has a significant impact on the healthcare‐associated infections in critical care unit. For nursing professionals, the high level of occupational burnout may result in absenteeism and a high job turnover rate, both of which have a negative impact on the quality care (Nowacka et al., 2018). Similarly, Cimiotti et al. (2012) also discovered that nurse burnout is significantly associated with urinary tract infection and surgical wound infection. A study by Baier et al. (2018) demonstrated a significant positive association between burnout, the safety outcomes injury and safety compromising behaviour for the emergency medical service workers in Germany. Given the above evidence, to our knowledge, studies about the burnout of novice nurses are lacking although they are regarded as the important resource of nurse team and are directly related to the nursing care quality of hospitals.

Mobile phone has become an indispensable part of our life. Study on the health issues related to mobile phone addiction and occupational burnout of novice nurses may contribute to provoke the continuing concerns about the patient safety. Given the serious consequences of mobile phone addiction and burnout on novice nurses and patients, the study aimed to describe mobile phone addiction, nursing adverse events, occupational burnout of novice nurses and ascertain the relationships among these variables, so as to provide a sound scientific evidence to design targeted intervention strategies.

## 3 THE STUDY

### 3.1 Aim

The study was carried out to describe mobile phone addiction, nursing adverse events, professional burnout and the relationships of them among Chinese novice nurses.

### 3.2 Design

A cross‐sectional descriptive design was conducted.

### 3.3 Participants

Participants were novice nurses from five public hospitals located in Sichuan Province, China. Convenience sample was used to recruit study participants. The eligibility criteria were as follows: (1) only enrolled nurses, with no more than three years’ work experience; (2) aged under 28; and (3) those who provided direct patient care currently and consented to take part in the survey.

### 3.4 Data collection

According to the eligibility criteria, nurse leaders from each hospital could recruit nurses. Those nurses who were interested in taking part in could contact the head researcher. The data were collected by five trained research assistants from January–February 2020. A total of 400 participants from five public hospitals filled in the questionnaires, out of which 366 questionnaires were collected back (91.5% response rate).

### 3.5 Ethical considerations

The study was approved by the Institutional Review Board of Xiangya Nursing School, Central South University. Before commencing the research, objective and content of this study were explained to the participants, as well as the possibility of leaving the study at any time. All the participants were reminded that their participation was voluntary and anonymous, and their information was confidential.

### 3.6 Data analysis

The statistical analysis software used in the study was SPSS 22.0 (SPSS Inc., Chicago, IL, USA). Continuous variables were described as means with standard deviations and categorical variables were presented as frequencies with percentages. An independent *t* test was used to compare the occurrence of nursing adverse events and scores of mobile phone addiction. The relationships between novice nurses’ mobile phone addiction and burnout were analysed using Pearson's correlation analysis. *p* Value < .05 was considered statistically significant (two‐tailed).

### 3.7 Validity and reliability

Mobile Phone Addiction Index (MPAI) was used to measure the level of mobile phone addiction of the participants. The scale consisted of 17‐item questionnaire and had 4 subscores including inability to control carving, feeling anxious and lost, withdrawal and productivity loss which was developed by Leung (2008) and revised to the Chinese version by Huang (2014). The items were assessed on a five‐point scale and the total scores range from 17–85, with a higher scores representing more severity for addiction symptom. The reliability of that scale of Cronbach's α is 0.91. Cronbach's α in the current study was 0.87.

Maslach Burnout Inventory Human Services Survey (MBI‐HSS) was used to measure the nurses’ burnout. It included three separate subscales, that is emotional exhaustion, depersonalization and personal accomplishment. The survey consisted of 22 questions. Each subcategory was scored on a Likert 7‐score scale (0 = never, 6 = daily). That instrument was developed by Maslach et al. (1986). The validity and reliability study of the Chinese version was conducted by Chen et al. (2000). The Chinese version scale has a good reliability and validity. The emotional exhaustion and depersonalization subscores indicating a high level were more than 26 and 9, respectively. For the personal accomplishment, a score between 0–34 meant occupational burnout. In the present study, Cronbach's α was 0.84.

## 4 RESULTS

### 4.1 Sample characteristics

Among the 366 participants, 18 (4.9%) were male and 348 females (95.1%). The mean age of the participants was 23.03 (*SD* 1.59), ranging from 20–28 years old. The other data are shown in Table 1

**Table 1:** Sample socio‐demographic characteristics (*N* = 366)

Variables	*n*	%	Mean	*SD*
Gender				
Female	348	95.1	‐	‐
Male	18	4.9	‐	‐
Age (years)			23.03	1.59
Marital status				
Single	325	88.8	‐	‐
Married	41	11.2	‐	‐
Education				
Diploma	8	2.2	‐	‐
Associate degree	306	83.6	‐	‐
Bachelor degree	52	14.2	‐	‐
Only child in the family				
Yes	117	32	‐	‐
No	249	68	‐	‐

### 4.2 Mobile phone addiction and nursing adverse events

The nursing adverse events of 366 participants were presented in Table 2. Significant higher mobile phone addiction scores were found in those with wrong medication (*p* = .002), patient fall with injury (*P *< 0.001), ulcer pressure (*p* = .002), nosocomial infection (*p* = .028) and unplanned extubation event (*P *< 0.001) and those without (Table 2).

**Table 2:** Mobile phone addiction scores according to the occurrence of wrong medication, ulcer pressure, patient fall, nosocomial infection and unplanned extubation (across a one‐year period) (*N* = 366)

Variables	*N* (%)	Mobile phone addiction Mean (*SD*)	*t*	*p*
Wrong medication			−3.62	<.001
No	300(81.97)	34.45 (10.20)		
Yes	66(18.03)	39.46 (10.04)		
Ulcer pressure			−3.09	.002
No	321(87.70)	34.73 (10.09)		
Yes	45(12.30)	39.77 (11.12)		
Patient fall with injury			−3.06	.002
No	300(81.97)	34.58 (10.17)		
Yes	66(18.03)	38.84 (10.43)		
Nosocomial infection			−2.20	.028
No	330(90.16)	34.96 (10.11)		
Yes	36(9.84)	38.94 (11.80)		
Unplanned extubation			−3.58	<.001
No	252(68.85)	34.07 (10.24）		
Yes	114(31.15)	38.19 (10.01）		

### 4.3 Burnout

The mean total scores of occupational burnout among 366 novice nurses were 62.25 (*SD* 14.19). 60.9% of participants (*N* = 223) presented a high emotional exhaustion score 29.31(*SD* 8.81). Analysis of the depersonalization subscale showed that 271 nurses (74%) presented high scores. As far as the personal achievement was concerned, 95.4% of the nurses presented a low sense of personal achievement 20.09 (*SD* 7.76) (Table 3).

**Table 3:** Occupational burnout scores (*N* = 366)

Dimensions	*N* (%)	Mean	*SD*
Emotional Exhaustion (EE)		29.31	8.81
Low	43 (11.75)	‐	‐
Average	100 (27.32)	‐	‐
High	223 (60.93)	‐	‐
Depersonalization (DEP)		12.85	4.68
Low	22 (6.11)	‐	‐
Average	73 (19.95)	‐	‐
High	271 (74.04)	‐	‐
Personal Achievement (PA)		20.09	7.76
Low	10 (2.73)	‐	‐
Average	7 (1.91)	‐	‐
High	349 (95.36)	‐	‐
Total score		62.25	14.19

### 4.4 Relationship between mobile phone addiction level and occupational burnout of novice nurses

Significant positive correlations were found between withdrawal (*r* = .311, *r* = .260), productivity loss (*r* = .331, *r* = .298), inability to control carving (*r* = .216, *r* = .267), feeling anxious and lost (*r* = .268, *r* = .253) and emotional exhaustion and depersonalization. There was no correlation between all the subscales of mobile phone addiction and personal achievement (Table 4).

**Table 4:** Correlation between mobile phone addiction and burnout (*N* = 366)

	Emotional exhaustion (EE)	Depersonalization (DEP)	Personal achievement (PA)
Inability to control carving	0.216*	0.267*	−0.021
Feeling anxious and lost	0.268*	0.253*	0.031
Withdrawal	0.311*	0.260*	−0.026
Productivity loss	0.331*	0.298*	0.036

* *P *< .01.

## 5 DISCUSSION

The mean score for the mobile phone addiction was 35.35 (*SD* 10.34). This study identified the mobile phone addiction levels of the novice nurses were slightly lower compared with the existing study (Leung, 2008). It is possibly because that college life may provide university students with much time and freedom and spend most of their time in the virtual world (Cerit et al., 2018). The results of this study supported the excess mobile phone use have a negative effect on the patient safety including nursing adverse events and low‐quality care. Novel findings were that the mobile phone addiction levels of novice nurses who had adverse events were observed to be significantly higher than those without those type events across a one‐year period. Likewise, Pucciarelli et al. (2018) reported that the mobile phone misuse can endanger the patient safety including clinical errors due to nurses' distraction and interruptions. Ustun et al. (2012) also observed that the healthcare providers’ mobile phones are potential agent of microbial cross‐contamination between hospital and community, which can lead to nosocomial infection. The results in this study are in accordance with a cross‐sectional survey performed by McBride et al. (2015), which showed that 78.1% (644/825) of registered nurses frequently use their mobile phones for non‐work‐related activities in clinical working environment and the potential distraction of mobile phone will be a threat to the patient safety. Based on those findings, it was concluded that the regulating mobile phone use novice nurses and reducing their mobile phone addiction levels are essential to ensure the patient safety and nurses’ health as well.

This study demonstrated that the percentages of novice nurses with a high level of emotional exhaustion (60.93%), high depersonalization (74.04%) and diminished sense of personal achievement (95.36%) are significantly higher than those of the previous studies (Miranda‐Ackerman et al., 2019; Poghosyan et al., 2009). Recently Yektatalab et al. (2019) examined 250 nurses working in Iranian health facilities and the percentages of the above three burnout subscales are 25.2%, 12.4% and 52%, respectively. Conversely, another study conducted on orthopaedic nurses in Italy, revealed that the incidence of high level emotional exhaustion (66%) and depersonalization (83%) are much higher than the results of this study, but the rate of diminished sense of personal achievement (88%) is lower (Martinelli et al., 2019). There is an obvious difference in the prevalence of three dimensions, which may be caused by the small sample size (95 nurses) in the Italian study. Besides that, the samples are only composed of orthopaedic nurses. Moreover, by comparing with other European countries, this study showed the prevalence of occupational burnout (52.46%) is much higher than Netherlands (10.23%) And Poland (40.20%), but much lower than Greece (78.10%) (Aiken et al., 2012). Additionally, this study shows higher scores of the burnout subscales than previous studies (Chen et al., 2019; Van Bogaert et al., 2010). Compared with most existing research results, the prevalence of severe occupational burnout among novice nurses in this study is high. One probable reason for our findings on the burnout is that novice nurses are in a potent transition and face many challenging situations (Ten Hoeve et al., 2018oeve et al., 2018). Furthermore, the highly prevalent burnout may be mainly caused by the work pressure, time irregularity, imprecise expectations and the soaring demand for medical service but lack of medical personnel and the unreasonable distribution of human resources in quantity and structure.

Another main finding of this study revealed that there was also a positive relationship between the mobile phone addiction level and burnout of the novice nurses (*r* = .33, *P *< .01). However, interestingly, the personal achievement was irrelevant to the mobile phone addiction level. To the best of our knowledge, the present study was the first to show the correlation between the level of mobile phone addiction and burnout in novice nurses. This study obtained a similar result to that from the USA. The study on a group of 385 osteopathic medical students performed by Brubaker et al. (2020) in 2018 detected that higher smartphone addiction scores are associated with higher emotional exhaustion and depersonalization but are not related with the personal achievement. Hence, the mobile phone addiction may have connection with the feeling of exhaustion by the work and impassive emotions towards others. The mental mechanism of the relationship between mobile phone addiction and burnout has not been interpreted explicitly and therefore, further studies are required with larger size and with participants of different occupations.

Lastly, this study proposed the issue of mobile phone addiction and burnout as a key point and the findings of this research can be applied to the novice nurses in the clinical environment. In addition, on the one hand, with regard to the mobile phone addiction, early intervention and detection for novice nurses are crucial because novice nurses who abandoned themselves to mobile phones may bring great threats to the patient safety due to distraction. On the other hand, as to high occupational burnout, it is also very prominent for managers to take measures to relieve the level in job burnout of novice nurses and increase the sense of personal achievement.

## 6 CONCLUSIONS

This study observed the differences in the mobile phone addiction levels between the novice nurses with and without nursing adverse events, which can produce serious consequences in terms of the patient safety and economic costs. Therefore, it is necessary to handle the immediate issue of dependence on the mobile phone and develop the relevant strategies for improving the patient care quality and ensuring the patient safety. Besides that, this study also identified high levels of burnout in novice nurses and further research is needed to more accurately assess the prevalence of that phenomenon. Ultimately, this study demonstrated that there was a correlation between the mobile phone addiction and burnout. Our research expanded the literature to include mobile phone addiction and burnout in novice nurses. In this sense, the role of mobile phone in nursing administration and education, especially as an assistance or obstacle to patient safety and burnout prevention, is an additional research field.

## 7 STUDY LIMITATIONS

Limitations of this study included the cross‐sectional design, convenience sample and participants’ self‐reported data. The cross‐sectional design of the study was not the best way to make a causal statement about the association between mobile phone addiction and burnout. Convenience sample led our results to lack generalizability to other populations. The study was conducted by self‐report questionnaire and limited the results. To get more details and conduct a comprehensive analysis, in‐depth interviews with novice nurses and nurse managers are necessary.

## CONFLICT OF INTEREST

The authors declare no potential conflicts of interest.

## AUTHOR CONTRIBUTIONS

HM wrote the manuscript and conducted statistical analysis and interpretation; JQH was in charge of the study concept and design; and JMZ and YZ were responsible for acquisition of data. All authors revised the final manuscript.

## Data Availability

The data used to support the findings of this study are available from the corresponding author upon request.
